# *Cereus sinensis* Polysaccharide and Its Immunomodulatory Properties in Human Monocytic Cells

**DOI:** 10.3390/md15050140

**Published:** 2017-05-18

**Authors:** Junwen Wu, Xuefei Zhou, Min Zhang, Yun Yao, Juanjuan Han, Kehai Liu

**Affiliations:** Department of Biopharmaceutics, College of Food Science and Technology, Shanghai Ocean University, Shanghai 201306, China; 15800711801@163.com (J.W.); zhouxuefeibest@163.com (X.Z.); 13122223561@163.com (M.Z.); m150208413@st.shou.edu.cn (Y.Y.); jjhan1409@163.com (J.H.)

**Keywords:** *Cereus sinensis*, polysaccharide, THP-1 cells, immunomodulatory property

## Abstract

In this study, the extraction conditions of the crude polysaccharide from *Cereus sinensis* were optimized by response surface methodology. The optimum extraction conditions were: a ratio of raw material to water volume of 1:80 (g/mL); an extraction temperature of 72 °C; and an extraction time of 3 h. Then, a purified polysaccharide named *Cereus sinensis* polysaccharide-1 (CSP-1) was obtained from the crude polysaccharide by the Diethylaminoethyl cellulose-52 (DEAE-52) cellulose chromatography column and Sephadex G-100 column. The molecular weight and monosaccharide composition of CSP-1 was determined through Gel Permeation Chromatography (GPC) and Gas Chromatography–Mass Spectrometer (GS–MS), respectively. The results showed that CSP-1 with an average molecular weight of 56,335 Da was composed of l-(−)-Fucose, d-(+)-Mannose, d-Glucose and mainly possessed 1→2, 1→2, 6, 1→4, and 1→4, 6 of glycosyl linkages. The immunomodulatory activities of CSP-1 were also evaluated using lipopolysaccharide (LPS)-induced human monocytic (THP-1) cells. The results demonstrated that CSP-1 dose-dependently protected against LPS-induced toxicity, and CSP-1 significantly inhibited the Toll-like receptor 4 (TLR-4) mRNA, myeloid differentiation factor 88 (MyD88) mRNA and tumour necrosis factor receptor-associated factor-6 (TRAF-6) mRNA expression of the LPS-induced THP-1 cells, as well as suppressing reactive oxygen species (ROS) generation.

## 1. Introduction

Marine organisms grew in the special environment of high salinity, high pressure, hypothermia, low light and oligotrophic qualities. As a result, the polysaccharides originating from marine organisms exhibited a unique structure and showed good prospects with immune regulation, anti-inflammation, anticoagulation, anti-virus, anti-tumor and so on [1]. Based on the origins, the polysaccharides were classified into three types: seaweed polysaccharides, marine microbial polysaccharides and marine animal polysaccharides [2]. At present, compared with marine animal polysaccharides, the research on seaweed polysaccharides and marine microbial polysaccharides were richer and more mature. In recent years, with the further development of marine resources, marine animal polysaccharides present unique properties, including good biocompatibility with human cells, low toxic side effects and significant physiological activities such as anti-tumor, anti-virus, anti-aging, anti-blood sugar, anticoagulant and so on [1,3,4], more and more attention has been focused on marine animal polysaccharides. For example, the polysaccharide from purple sea urchin shells showed anti-inflammatory activity by inhibiting RAW264.7 cell growth and releasing NO production [5], and a polysaccharide isolated from *Meretrix meretrix* Linnaeus presented immunological activity [6]. Previous studies also have demonstrated that *Cyclina sinensis*, *Hyriopsis cumingii* and other marine animal polysaccharides have exhibited anticancer activity [7].

As a marine animal, it has been recorded that sea anemone (Coelenterata, Anthozoa, Actiniaria) possess many pharmaceutical properties, including antitumor [8,9], antihypertensive, antibacterial, analgesic, and anti-inflammatory [10]. At the same time, sea anemone as a food has been accepted by more and more people. So far, attention has mainly been paid to polypeptides, proteins, the toxins structure and the field of biological activity of sea anemone; however, there are rarely studies done on polysaccharides of sea anemone [11,12]. The chemical components of *Zoanthus stephensoni* growing in the Xisha Islands of the South China Sea were studied by Zheng for the first time. Meanwhile, the polysaccharides extracted from these sea anemones were obviously effective in lowering blood pressure [13,14].

*Cereus sinensis* belongs to Actiniaria, *Hormathiidae Carlgren*, *Calliactis Verrill* [15], which is widely distributed in coastal waters of the Pacific and China’s Yellow Sea, Bohai Sea, East Sea, South Sea and so on. Furthermore, there are not currently not many studies on *Cereus sinensis*. For instance, the cytolytic toxins were preliminarily isolated from *Cereus sinensis* Verrill, the factors influencing cytolytic toxins were investigated [16], and the analysis on nutrients and non-volatile tastecompounds of *Cereus sinensis* was carried out [17]. However, the research on *Cereus sinensis* polysaccharides and their biological activities has not been reported. In this study, we investigated the partial characterization of a purified polysaccharide CSP-1 isolated from *Cereus sinensis* and evaluated its immunomodulatory activites in vitro.

## 2. Results

### 2.1. Optimization of the Polysaccharide Extraction

The polysaccharide of *Cereus sinensis* was extracted through “the hot water extraction and alcohol precipitation” method, which was optimized by response surface methodology. The three parameters (the solid:liquid ratio, the extraction temperature and the extraction time) were investigated in this experiment (Table 1). As a result, the optimum extraction condition of *Cereus sinensis* polysaccharide is a ratio of raw material to water volume of 1:80 (g/mL), an extraction temperature of 72 °C and an extraction time of 3 h (Table 2).

### 2.2. Isolation and Purification of Polysaccharide

**Figure 1:**
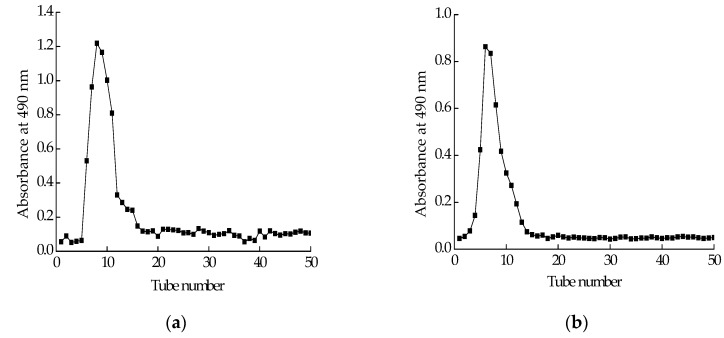
Purification of polysaccharide: (**a**) elution profile of crude polysaccharide extracted from *Cereus sinensis* on a DEAE-52 (Diethylaminoethyl cellulose) anion-exchange column; (**b**) elution profile of fraction from A on Sephadex G-100 column.

Polysaccharide isolated from *Cereus sinensis* was first eluted through a DEAE-52 (Diethylaminoethyl cellulose) anion-exchange column to yield one peak (Figure 1a). The elution product was collected, dialyzed and lyophilized to obtain a polysaccharide named CPS. CPS was further purified by a Sephadex G-100 column. The elution curve was shown in Figure 1b. The elution product was collected, dialyzed and lyophilized to obtain a purified polysaccharide named CPS-1.

### 2.3. Molecular Weight and Monosaccharide Composition of CPS-1

Gel Permeation Chromatography (GPC) was applied to determine the average molecular weight (MW) of CSP-1, using polyethylene glycol (PEG) as the standard to establish the calibration curve. The result indicated that its average molecular weight was 56335 Da (Table 3). The monosaccharide composition of CSP-1 was analyzed by Chromatography-Mass Spectrometer (GC-MS). The result showed that CSP-1was composed of l-(−)-Fucose, d-(+)-Mannose, and d-Glucose (Table 4).

### 2.4. Periodate Oxidation of CSP-1

The results showed that 0.963 mol of periodate was consumed per mole of sugar and 0.127 mol formic acid was produced. The formation of formic acid suggested the presence of pyranohexose in 1→ or 1→6 linked forms in 12.7%. As the amount of periodate consumption was more than twofold the amount of formic acid produced, linkages that only consumed periodate without formic acid production were therefore deduced to exist as 1→2, 1→2, 6, 1→4, and 1→4, 6 forms, which occupied 70.9% of the total glycosyl linkages. The ratio of other linkages as 1→3-linked forms was 16.4%, which did not consume periodate.

### 2.5. Effects of CPS-1 on Cell Viability

**Figure 2:**
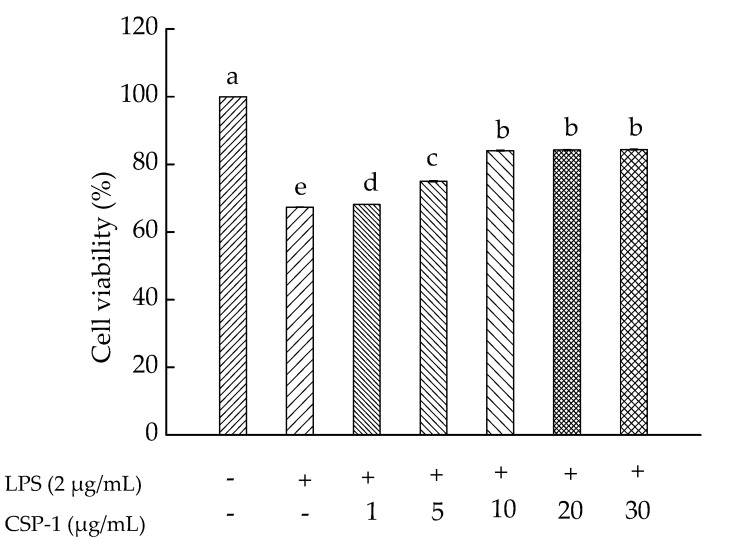
Influences of *Cereus sinensis* polysaccharide on cell viability. Cells were treated with LPS (2 μg/mL) for 24 h in the absence or presence of CSP-1 at different concentrations (1, 5, 10, 20 and 30 μg/mL). MTT (3-(4,5-dimethylthiazol-2-yl)-2,5-diphenyltetrazolium bromide) assay was conducted to measure cell viability. Values are mean ± SD (standard deviation) (*n* = 3); bars with the same letter being accepted, suggesting no significant differences between groups when the value of *p* was < 0.05 in accordance with Duncan’s multiple range test. “−” and “+” denotes no addition and addition of sample, respectively.

As shown in Figure 2, the effect of CSP-1 on lipopolysaccharide (LPS)-induced human monocytic (THP-1) cell viability was investigated. Results showed that CSP-1 concentration-dependantly prevented LPS-induced toxicity in the THP-1 cells. When the concentration was achieved at 10 μg/mL or more, CSP-1 was able to protect the THP-1 cells against LPS-induced toxicity. Thus, CSP-1 at this concentration was selected for further studies of immunomodulatory properties.

### 2.6. CPS-1 Inhibited the LPS-Induced ROS Formation

**Figure 3:**
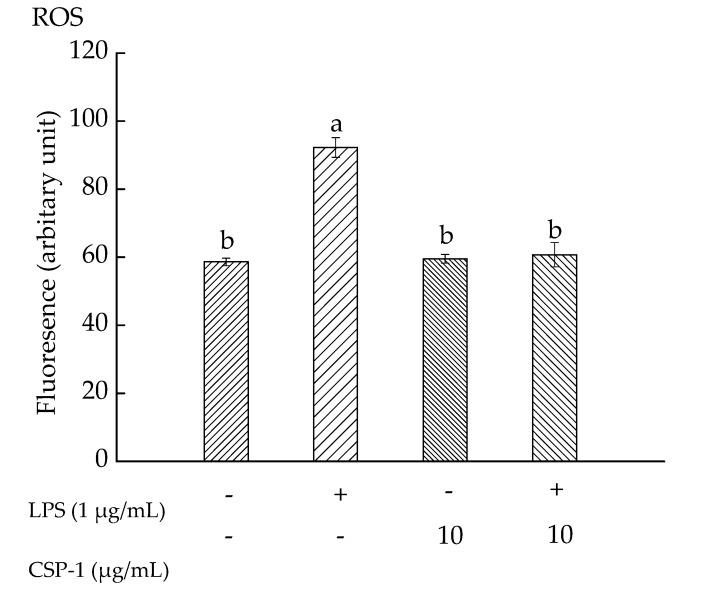
Influences of CSP-1 on reactive oxygen species (ROS) generation. Cells were treated with LPS (1 μg/mL) for 24 h in the absence or presence of CSP-1 (10 μg/mL), followed by adding 10 μM DCFH-DA to incubate for 30 min. Values are mean ± SD (*n* = 3); bars with the same letter were accepted as suggesting no significant differences between groups when value of *p* was < 0.05 in accordance with Duncan’s multiple range test. “−” and “+” denotes no addition and addition of sample, respectively.

Exposure of THP-1 cells to LPS (1 μg/mL) alone resulted in increasing the mean fluorescence intensity observably, from 58.63 ± 1.12 (a.u) to 92.25 ± 2.89 (a.u). However, CSP-1 (10 μg/mL) treatment caused the fluorescence intensity to decrease to 60.76 ± 3.58 (a.u) in LPS-stimulated cells (Figure 3). These results demonstrated that LPS (1 μg/mL) treatment gave rise to a significant increase in ROS level, but CSP-1 treatment (10 μg/mL) significantly stopped the ROS generation in LPS-stimulated THP-1 cells.

### 2.7. PSCPL Influenced the TLR-4, MyD88 and TRAF-6 Signal Transduction Pathways

**Figure 4:**
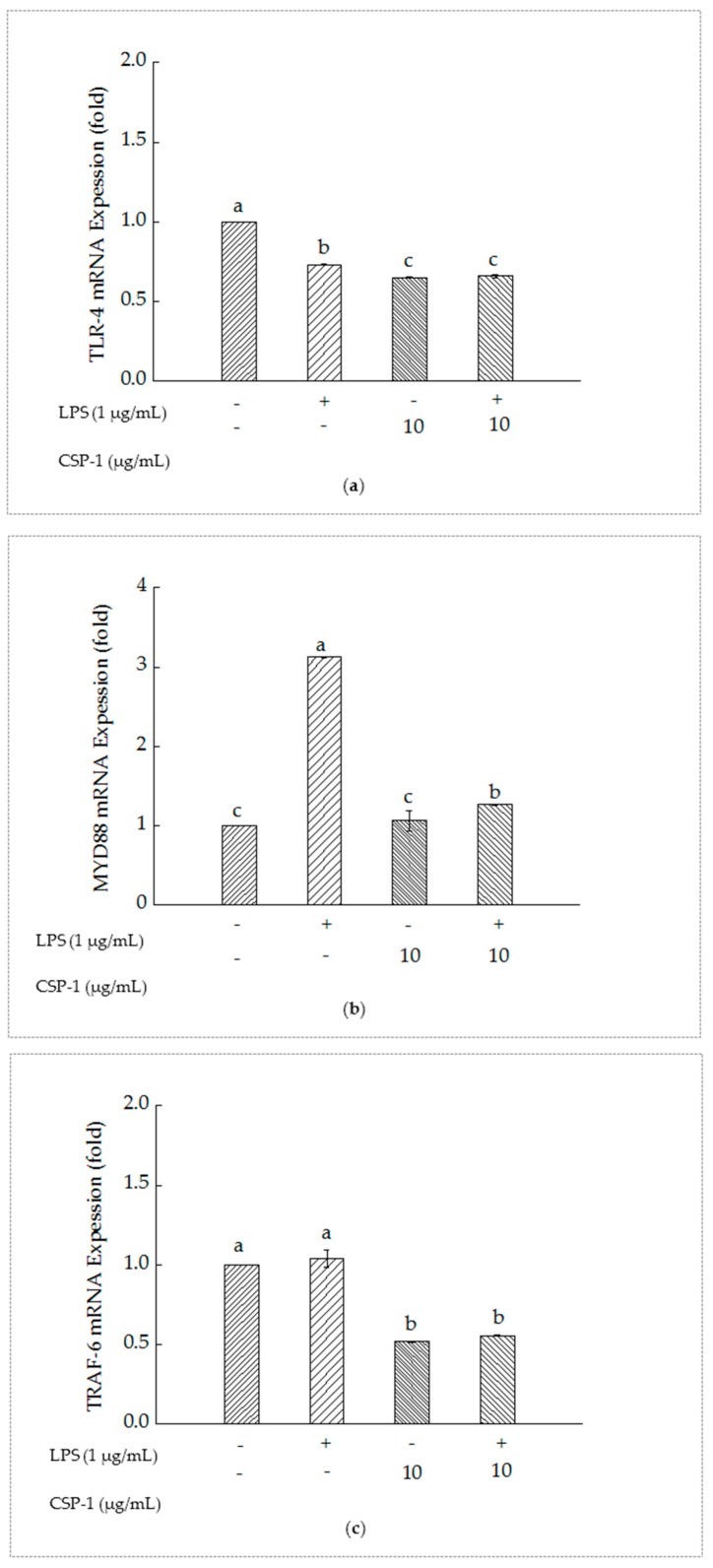
Influences of CSP-1 on TLR-4 mRNA (**a**), MyD88 mRNA (**b**) and TRAF-6 mRNA (**c**) expression. Cells were stimulated with LPS (1 μg/mL) for 24 h in the absence or presence of CSP-1 (10 μg/mL). Total RNA was extracted and the content of gene expression was determined by real-time RT-PCR. Values are mean ± SD (*n* = 3); bars with the same letter were accepted as suggesting no significant differences between groups when value of *p* was < 0.05 in accordance with Duncan’s multiple range test.

As shown in Figure 4a, compared with the un-treated cells, Toll-like receptor (TLR-4) mRNA expression of the cells with LPS treatment reduced significantly. Furthermore, simultaneous exposure of LPS and CSP-1 showed a remarkable difference in the expression of TLR-4 mRNA from those treated with LPS alone. The results suggested that CSP-1 could downregulate the expression of TLR-4 mRNA in LPS-stimulated THP-1 cells.

As showed in Figure 4b, MyD88 mRNA expression of the THP-1 cells increased significantly as a result of LPS treatment. However, CSP-1 treatment led to remarkable reduction of MyD88 mRNA expression in LPS-induced THP-1 cells, which indicated that CSP-1 could downregulate the MyD88 mRNA expression of the LPS-induced THP-1 cells.

As showed in Figure 4c, TRAF-6 mRNA expression of the THP-1 cells showed no difference from those treated with LPS only. When CSP-1 was added to the LPS-stimulated THP-1 cells, the expression of Tumour necrosis factor receptor-associated factor (TRAF-6) mRNA declined observably. This demonstrated that the TRAF-6 mRNA expression of the LPS-induced THP-1 cells might be downregulated with CPS-1 treatment.

## 3. Discussion

In this study, the polysaccharide was isolated and purified from *Cereus sinensis* for the first time and the immunomodulatory activity was investigated using LPS-induced THP-1 cells. The effect of CSP-1 on RAW264.7 cells had already been investigated using alamar blue assay, and results showed that CSP-1 (1–30 μg/mL) exhibited no cytotoxicity to these cells. The experiment demonstrated that CSP-1 (10 μg/mL or more) could protect THP-1 cells against LPS-stimulated toxicity. The ROS generation, TLR-4 mRNA expression, MyD88 mRNA expression and TRAF-6 mRNA expression of LPS-induced THP-1 cells were significantly inhibited by adding 10 μg/mL CSP-1. A polysaccharide from sea anemone has been reported to possess immune activity in vitro. For example, the polysaccharide isolated from *Edwardsia ipunculoides* exhibited immune activity by promoting the concentration of TNF-α in THP-1 cells [12]. TNF-α is a cytokine produced by foreign antigen stimulated-macrophages, which can directly kill most of the tumor cells, as well as promoting wound healing, angiogenesis and other effects [18,19].

Exposure of THP-1 cells to LPS resulted in activation of the TLR-4, which led to the activation of signaling cascades, including MyD88 and TRAF-6 [20]. At the same time, it was proposed that simultaneous exposure of THP-1 cells to LPS and compounds, combined with gene expression analysis, was a useful in vitro screening tool to select inflammation modulating compounds [21]. Furthermore, previous studies showed that chronic inflammatory diseases could be treated effectively by modulating TLR signaling pathways [22,23]. Astragalus polysaccharide inhibited LPS-induced cardiomyocyte hypertrophy in rats through the TLR4/NF-κB (nuclear factor) signal transduction [24]. The dysregulation of TLR-4 signaling pathway had a close relationship with development and progress of various diseases, such as nephrotic syndrome, nephritis, renal insufficiency, hypothyroidism [25], systemic lupus erythematosus [26], diabetes [27] and so on. As a result, we investigated the effects of CSP-1 on LPS/TLR-4 signal pathways and the adaptor protein of this signal pathway in THP-1 cells, including MyD88 and TRAF-6, to explore its immunomodulatory mechanism. In our study, CSP-1 treatment downregulated TLR-4 mRNA expression in LPS-stimulated THP-1 cells. Inhibition of TLR-4 expression was a kind of negative regulator for TLR-4 signaling [28]. Quantitative RT-PCR and luciferase reporter gene experiments confirmed that the expression of TLR-4 was inhibited after miRNA *lethal-7i* at post-transcriptional level interacted with the 3′-untranslated region of TLR-4 mRNA [29,30]. It was possible that inhibition of TLR-4 mRNA expression linked to TLR-4 expression suppression. The MyD88-dependent pathway belonged to TLR-4 signaling [20]. MyD88 was an immediate adaptor molecule, which was recruited by activated TLR-4, and was critical for triggering the activation of signaling cascades, including IRAK-1(interleukin receptor) and IRAK-4 [31,32]. MyD88 formed a complex with TLR-4, IRAK1, and IRAK-4, and then IRAK1 was phosphorylated and separated from the complex [33,34]. TRAF-6 was another adaptor protein that was recruited and activated by phosphorylated IRAK1 and played an essential role in activating transforming growth (TAK1) [20]. Subsequently, TAK1 recruited and activated the complex of NF-κB and inhibitor of nuclear factor (IKK). Hereafter, Inhibitor of nuclear factor kappa B (IκB) was phosphorylated, ubiquitinated and degradated. It led to NF-κB separating from IκB and entering the nucleus, followed by initiating or enhancing the inflammatory genes transcription and promoting expression of inflammatory factors and inflammatory chemokines, such as tumor necrosis factor-α (TNF-α) and Interleukin-12 (IL-12) [35,36]. TAK1 also activated the mitogen-activated protein kinase (MAPK) signaling pathway, which led to the activation of subgroups extracellular signal-regulated kinase (ERK), p38 mitogen-activated protein kinase and C-jun n-terminal kinase (JNK), followed by formation of transcription factor activator protein-1 (AP-1), regulation of IL-1, IL-6 and TNF-α and other inflammatory factors of transcription in the end [37,38]. Consistent with the effects of *Phellinus linteus* polysaccharides on LPS-stimulated THP-1 cells [39], CSP-1 treatment significantly inhibited ROS generation and led to downregulation of MyD88 mRNA and TRAF-6 mRNA expression in LPS-induced THP-1 cells. It was probable that expression of MyD88 mRNA and TRAF-6 mRNA decreased in the MyD88-dependent signaling pathway and ROS generation reduced in the TLR-4/NADPH oxidase/ROS signaling pathway as a result of TLR-4 signaling transduction weakening by inhibition of TLR-4 expression.

ROS was indispensable for activation of NF-κB and MAPKs [40]. It was reported that the increase of ROS production was a prerequisite for the activation of p38 MAPK in LPS-stimulated cardiomyocytes [41]. In addition, Wu showed that suppression of LPS-induced ROS generation with PSCPL treatment resulted in repressing the downstream signals of the TLR-4 pathways, such as cytokine production, NF-κB p65 activation, and so on [39]. In this study, ROS generation was suppressed by CSP-1, which might also attenuate the downstream signal in the TLR-4 signaling pathway.

It had been reported that attenuating TLR-4 signaling was a potential effective therapy for coronary artery disease [29], and the inhibition of the ROS generation, expression of TLR-4 mRNA, MyD88 mRNA and TRAF-6 mRNA in LPS-stimulated THP-1 cells with CSP-1 treatment is strong evidence for its potential immunomodulatory activities.

## 4. Materials and Methods

### 4.1. Samples and Materials

*Cereus sinensis* was purchased from Taizhou, Zhejiang Province, China. Roswell Park Memorial Institute (RPMI) 1640 medium and fetal bovine serum (FBS) were purchased from Invitrogen (Carlsbad, CA, USA). Lipopolysaccharide (LPS), 3-(4,5-dimethylthiazol-2-yl)-2,5-diphenyl tetrazolium bromide (MTT), dimethyl sulfoxide (DMSO), 2′,7′-dichlorofluorescin diacetate (DCFH-DA) and buffered solution (PBS) were purchased from Sigma-Aldrich (St. Louis, MO, USA). Sodium chloride, hydrochloric acid, sodium hydroxide, ethanol, acetone, pyridine, acetic anhydride trichloroacetic acid, trifluoroacetic acid (TFA), hydroxylamine hydrochloride, sodium periodate, ethylene glycol, phenolphthalein and trichloromethane were bought from Sinopharm Chemical Reagent Co., Ltd. (Shanghai, China).

### 4.2. Sample Preparation, Isolation and Purification of the Polysaccharide

Fresh sea anemones were soaked in 2% NaCl solution for two days to remove impurities. Then, the water of sea anemones surface was wiped with absorbent paper. Subsequently, the sea anemones were homogenized, followed by addition of equal volume acetone (overnight) to remove fats. After filtering with gauze, the residue was freeze-dried, sealed in the Ziploc bag and stored in the refrigerator at −20 °C. The response surface methodology was used to get the optimal extraction conditions of the crude polysaccharide from *Cereus sinensis* according to Table 1.

Degreased sea anemone powder was extracted with distilled water (1:80 solid/liquidratio) for 3 h at 72 °C. Then, the aqueous extracts were filtered twice and precipitated with three fold 95% (*v*/*v*) ethanol for 2 days at room temperature. Subsequently, the mixture was centrifuged at 4000 rpm for 20 min at 4 °C and precipitation was dried at 60 °C in constant temperature drying oven to obtain the *Cereus sinensis* crude polysaccharide. The crude polysaccharide (5 g) was dissolved in 100 mL distilled water; hereafter, supernatant was collected by centrifugation at 4000 rpm for 20 min at 4 °C and deproteinised by addition of 3% (*m*/*v*) trichloroacetic acid to a final concentration of 20% (*v*/*v*) overnight at 4 °C. After removal of the protein, the mixture was concentrated by rotator evaporator (50 rpm 60 °C) and dialyzed (3500D MWCO) against ultrapure water for 2 days and then freeze-dried (24 h) to yield the polysaccharide. Furthermore, 20 mg/mL polysaccharide solution (5 mL) was prepared and loaded onto a DEAE-52 cellulose chromatography column, and washed with water, followed by a linear gradient elution with an NaCl solution (0.1, 0.3 mol/L) at a flow rate of 0.5 mL/min. The polysaccharide content was determined by testing the absorbance of each fraction (6 mL) at 490 nm according to the phenol-sulfuric acid method. The fraction containing polysaccharide was combined, concentrated, dialyzed (3500D MWCO) and lyophilized. This polysaccharide was further purified by a Sephadex G-100 column, and eluted with 0.1 mol/L NaCl at a flow rate of 0.5 mL/min to yield a single peak (Figure 1b). The fraction was collected, concentrated, dialyzed (3500D MWCO) and lyophilized to get a purified polysaccharide.

### 4.3. Determination of the Molecular Weight of Polysaccharide

The molecular weight of polysaccharide was determined by GPC (Waters 2695 GPC, Manchester, UK), using three GPC columns of HR3, HR4 and HR5 column (7.8 mm × 300 mm), and a Waters 2414 refractive index detector. A fifty microliter sample was injected, and mobile phase consisting of PBS and NaN_3_ was used at a flow rate of 1 mL/min. Column temperature was set as 40 °C and operation time was 45 min. PEG was used as standard sample and established standard curve.

### 4.4. Analysis of Monosaccharide Composition

The monosaccharide composition of CSP-1 was detected using GS-MS. Furthermore, 10 μg CSP-1 was accurately weighed, and then 2 mL TFA (2 mol/L) was added to a sealed round-bottomed flask to hydrolyze in a 100 °C water bath for 6 h. Subsequently, the TFA was completely removed by rotating and evaporating at 40 °C under reduced pressure. The hydrolyzate was dissolved in 0.5 mL pyridine and reacted in a 90 °C water bath for 30 min with successive shaking after adding 10.0 mg hydroxylamine hydrochloride. After cooling to room temperature, 0.5 mL acetic anhydride was added and reacted in a 90 °C water bath for another 30 min with shaking. After cooling to room temperature, the solution was rotated and evaporated at 50 °C under reduced pressure. Hereafter, 1 mL chloroform was added and centrifuged at 4000 rpm for 5 min to obtain a supernatant for GS-MS analysis. The analysis was performed on an Agilent 7890A gas chromatograph (Agilent Technologies, Little Fall, NY, USA)equipped with a DB-5MS column (30 m × 0.25 mm × 0.25 μm) coupled with an Agilent 5975C mass spectrometer (Agilent Technologies, Little Fall, NY, USA). Eight monosaccharide standards (d-Glucose, d-(+)-Mannose, l-(+)-Rhamnose, d-(+)-Galactose, d-Xylose, l-(−)-Fucose, d-Allose and dl-Arabinose) were prepared using the same method. The operation was performed under the following procedure: injection temperature: 270 °C; injection volume: 1.0 μL; column temperature programmed as: 100 °C for 5 min, and then increased to 190 °C at 20 °C/min, 260 °C at 3 °C/min, and finally 300 °C at 10 °C/min keeping for 5 min; He (99.999%): 1.0 mL/min; no shunt; interface temperature: 270 °C; ion source temperature: 230 °C; four pole temperature: 150 °C; electron bombardment source: 70 eV; electron multiplier: 2153 V; scanning range: 33–550 amu; National Insititute of Standards and Technology (NIST) library.

### 4.5. Periodate Oxidation

In addition, 30 mg of CSP-1 was oxidized with 30 mL 15 mmol/L NaIO_4_ and kept in the dark for nearly 48 h at 4 °C. The absorbance was measured at 223 nm every 6 h until the consumption of NaIO_4_ reached a constant value. Subsequently, ethylene glycol (1 mL) was added to samples (2 mL) to prevent the reaction. The amount of formic acid produced was titrated with 5 mmol/L NaOH.

### 4.6. Cell Culture

THP-1 was obtained from the cell bank of the Chinese Academy of Science (Shanghai, China). Cells were cultured in RPMI-1640 medium with 10% heat-inactivated FBS and incubated at 37 °C in a humidified 5% CO_2_ atmosphere.

### 4.7. MTT Assay

The cytotoxicity of polysaccharide was measured by MTT assay. THP-1 cells were distributed to each well of a 96-well plate for 24 h and each well contained 100 μL cell suspension. Furthermore, 100 µL serum-free media with final concentrations of the 2 μg/mL LPS or 2 μg/mL LPS plus 1 μg/mL, 5 μg/mL, 10 μg/mL, 20 μg/mL, and 30 μg/mL of CPS-1 were added to each cell well, respectively. After incubating the plate for 24 h with 5% CO_2_ at 37 °C, cells were collected by centrifugation at 1000 rpm for 5 min and washed with buffered solution (PBS) before adding 20 μL 5 mg/mL sterilized MTT solution and 180 µL of fresh growth medium, and kept at 37 °C for 4 h. Subsequently, the MTT/growth medium was removed by centrifugation at 1000 rpm for 5 min, replaced by 150 µL DMSO and kept for 10 min with gentle vortexing at room temperature to dissolve blue formazan formed by living cells. The absorbance value was measured at 570 nm with a microplate reader.

### 4.8. Intracellular ROS Assay

THP-1 cells were distributed to a 24-well plate for 24 h and each well contained 900 μL cell suspension. Then, 100 μL serum-free media with final concentrations of the 1 μg/mL LPS, 10 μg/mL CPS-1 or 1 μg/mL LPS plus 10 μg/mL CPS-1 were added to each cell well, respectively. After incubating the plate for 24 h with 5% CO_2_ at 37 °C, cells were collected by centrifugation at 1000 rpm for 5 min and washed with PBS. Subsequently, cells were treated with 10 μM of DCFH-DA and then incubated in the dark for 30 min with 5% CO_2_ at 37 °C. At last, cells were washed with PBS twice and placed on ice to be tested by flow cytometry.

### 4.9. Quantitative Reverse Transcriptase Polymerase Chain Reaction (RT-PCR) Analysis

Total RNA from THP-1 cell was isolated using RNAiso Plus Code NO. 9108/9109, TaKaRa Bio Inc., Dalian, Liaonin, China). Subsequently, total RNA was reverse transcribed to cDNA using PrimeScript™ RT reagent Kit with gDNA Eraser (Code NO. PR047A, TaKaRa Bio Inc., Dalian, Liaonin, China). The Applied Biosystems 7500 Real Time PCR System (Applied Biosystems, Foster City, CA, USA) was applied to quantify the relative content of mRNA. The PCR conditions of initial denaturation were 1 rep at 95 °C for 30 s, followed by 40 reps at 95 °C for 5 s, 60 °C for 34 s, and 1 rep at 95 °C for 15 s, 60 °C for 1 min, and 95 °C for 15 s. The primers used were human GAPDH sense primer, 5′-AAA TCC CAT CAC CAT CTT CC-3′; antisense primer, 5′-GCA GAG ATG ATG ACC CTT T-3′; human TLR-4 sense primer, 5′-ATG CCT GTG CTG AGT TT-3′; antisense primer, 5′-CTC TAC CAT ACT TTA TGC AGC C-3′; human MyD88 sense primer, 5′-CTA GGT GGG AAA GTC CCA TCA-30′; antisense primer, 5′-TCT TCC TCT CTC TGT GCT TCA TTA-3′; and human TRAF-6 sense primer, 5′-TCC TTG CCC TGTTCT CAA T-3′; and antisense primer, 5′-GCA TGG AAC GTG TGGAT-3′.

### 4.10. Data Statistics

Data were expressed as mean ± standard deviation (SD) of three repeated experiments. SPSS Statistics 17.0 (SPSS, Chicago, IL, USA) was used to calculate values according to one way analysis of variance (ANOVA) and Duncan’s multiple range tests. *p*-value of less 0.05 was considered to be significantly different.

## 5. Conclusions

In this paper, the optimal extraction conditions of *Cereus sinensis* polysaccharide were investigated using response surface methodology and three parameters (ratio of material to liquid, extraction temperature and extraction time) were studied. The results showed that the optimum extraction condition was as follows: a ratio of material to water volume of 1:80 (g/mL); extraction temperature 72 °C and extraction time of 3 h. The average molecular weight of CSP-1 was 56335 Da, it was composed of l-(−)-Fucose, d-(+)-Mannose, d-glucose and mainly possessed 1→2, 1→2, 6, 1→4, and 1→4, 6 glycosyl linkages. The LPS-induced toxicity of THP-1 cells was eliminated by adding to CSP-1 at 10 μg/mL or more. Furthermore, CSP-1 exhibited immunomodulatory properties by inhibiting ROS generation and downregulating TLR-4 mRNA, MyD88 mRNA and TRAF-6 mRNA expression in LPS-induced THP-1 cells.

## Figures and Tables

**Table 1:** Experimental design and results of response surface methodology.

Number	Solid:Liquid Ratio (g/mL)	Time (h)	Temperature (°C)	Extraction Yield (%)
1	1:90	3	60	21.22
2	1:80	2	60	18.67
3	1:90	4	70	23.20
4	1:70	3	80	21.05
5	1:80	4	80	22.15
6	1:80	4	60	22.30
7	1:70	3	60	20.20
8	1:80	3	70	23.80
9	1:80	3	70	23.76
10	1:80	3	70	23.80
11	1:80	3	70	23.78
12	1:90	3	80	23.34
13	1:70	2	70	20.05
14	1:80	2	80	22.05
15	1:70	4	70	20.98
16	1:80	3	70	24.50
17	1:90	2	70	20.45

**Table 2:** Variance analysis and significance test.

Source	Sum of Square	df	Mean Square	F Value	Prob > F	Significance
Model	44.85	9	4.98	72.93	<0.0001	significant
A-Solid:liquid ratio (g/mL)	4.4	1	4.4	64.32	<0.0001	
B-Time (h)	6.86	1	6.86	100.44	<0.0001	
C-Temperature (°C)	4.81	1	4.8	70.31	<0.0001	
AB	0.83	1	0.83	12.12	0.0103	
AC	0.4	1	0.4	5.9	0.0455	
BC	3.12	1	3.12	45.59	0.0003	
A^2^	7.1	1	7.1	103.97	<0.0001	
B^2^	8.96	1	8.96	131.16	<0.0001	
C^2^	5.83	1	5.83	85.28	<0.0001	
Residual	0.48	7	0.068			
Lack of fit	0.068	3	0.023	0.22	0.8767	not significant
Pure error	0.41	4	0.1			
Cor total	45.33	16				

**Table 3:** GPC analysis of CSP-1. Mn: Number-average molecular weight; M_W_: Weight-average molecular weight; M_P_: Peak molecular weight; M_Z_: Z-average molecular weight; M_Z_ + 1: Z + 1-average molecular weight.

Dist Name	Retention Time (min)	Adjusted RT (min)	Mn	M_W_	M_P_	M_Z_	M_Z_ + 1
	28.228	28.228	2505	56,335	3937	263,586	691,260

**Table 4:** GC-MS analysis of CSP-1.

Name	Retention Time	Type	Peak Width	Peak Area	Starting Time	End Time
l-(−)-Fucose	12.4	BB	0.048	13,501,531	12.219	12.489
d-(+)-Mannose	15.342	BV	0.04	1,077,806	15.203	15.4
d-glucose	15.488	VB	0.042	904,727	15.4	15.562

## Abbreviations

CSP	*Cereus sinensis* Polysaccharide
DEAE	Diethylaminoethyl cellulose
GPC	Gel Permeation Chromatography
GS–MS	Gas Chromatography–Mass Spectrometer
LPS	Lipopolysaccharide
THP-1 cells	Human monocytic cells
TLR-4	Toll-like receptor 4
MyD88	Myeloid differentiation factor 88
TRAF-6	Tumour necrosis factor receptor-associated factor-6
PEG	Polyethylene glycol
Mn	Number-average molecular weight
M_W_	Weight-average molecular weight
M_P_	Peak molecular weight
M_Z_	Z-average molecular weight
M_Z_+1	Z+1-average molecular weight
MTT	3-(4,5-dimethylthiazol-2-yl)-2,5-diphenyltetrazolium bromide
SD	Standard deviation
NF-κB	Nuclear factor kappa B
*let-7i*	Lethal-7i
IRAK	Interleukin receptor associated kinase
TAK	Transforming growth factor-activated Kinase
IKK	Inhibitor of nuclear factor kappa-B kinase
IκB	Inhibitor of nuclear factor kappa B
TNF-α	Tumor necrosis factor-α
IL	Interleukin
MAPK	Mitogen-activated protein kinase
EPK	Extracellular signal-regulated kinase
p38	p38 mitogen-activated protein kinase
JNK	c-Jun N-terminal kinase
AP	Activator protein
RPMI	Roswell Park Memorial Institute
NIST	National Insititute of Standards and Technology
